# Supracubital perineurioma misdiagnosed as carpal tunnel syndrome: case report

**DOI:** 10.1186/1471-2377-4-19

**Published:** 2004-11-21

**Authors:** Carsten Saft, Juergen E Andrich, Eva Neuen-Jacob, Gebhard Schmid, Ludger Schols, Georgios Amoiridis

**Affiliations:** 1 Department of Neurology, St. Josef Hospital, Ruhr-University Bochum, Gudrunstraße 56, 44791 Bochum, Germany; 2 Department of Neuropathology, Heinrich-Heine-University, Moorenstraße 5, 40225 Düsseldorf, Germany; 3 Department of Radiology, St. Josef Hospital, Ruhr-University Bochum, Gudrunstraße 56, 44791 Bochum, Germany; 4 Department of Neurology, Hertie-Institut für Klinische Hirnforschung, Hoppe-Seyler-Str 3, 72076 Tübingen, Germany; 5 Department of Neurology, School of Health Sciences, University of Crete, P.O. Box 2208, 71003 Heraklion Crete, Greece

## Abstract

**Background:**

Perineuriomas have been defined as tumorous lesions of the peripheral nerves which derive from perineurial cell proliferation and may be associated with abnormalities on chromosome 22.

**Case presentation:**

Three years after a painful cubital vein procaine injection, a 33 year-old man developed a median nerve lesion, initially diagnosed as carpal tunnel syndrome. Symptoms progressed despite appropriate surgery. Clinical and electrophysiological re-evaluation revealed a fusiform mass at the distal upper arm, confirmed by MRI. Immunohistochemical studies classified the tumor as a mixed perineurioma and neuroma.

**Conclusions:**

Perineurioma mixed with neuroma may potentially caused by the previous trauma or cytotoxic effects of procaine.

## Background

In the past years, there has been much confusion concerning the definition of the entity of rare focal lesions of the peripheral nerves since terms such as perineurioma, localized hypertrophic neuropathy, or hypertrophic neuritis have been used as synonyms [1-5]. According to the revised World Health Organization classification of tumors of the nervous system, however, perineuriomas have been defined as tumorous lesions of the peripheral nerves which derive from perineurial cell proliferation. They show strong immunoreactivity for the epithelial membrane antigen and may be associated with abnormalities on chromosome 22 [6-9].

In contrast, localized hypertrophic neuropathy has been defined as a distinct entity which is comprised of Schwann cell-onion-bulb formations, immunohistochemically stains strongly for protein S100, and is epithelial membrane antigen negative [9-11]. Localized hypertrophic neuropathy may be caused by a non-neoplastic undefined stimulus [12,13].

## Case presentation

Three years after a cubital procaine-HCL 0.05% injection had caused acute severe local pain radiating to his forearm and wrist, a 33-year old man complained about pain in his right hand. Two months later, he also suffered from numbness in the distribution of the right median nerve and wasting of thenar muscles. Furthermore a muscular atrophy in his right forearm was noted. Nerve conduction studies showed an increased distal latency in the median nerve, a very low thenar compound muscle action potential on median nerve stimulation, a reduced sensory conduction velocity of 39 m/s in the median nerve on thumb stimulation and no sensory nerve action potential on stimulation of the 2^nd ^and 3^rd ^fingers. Electromyography of the right biceps brachii muscle was normal. In the fibrillations, an increased duration and amplitude of motor unit potentials and reduced recruitment pattern were found in the right abductor pollicis brevis muscle. Forearm muscles were not investigated. A carpal tunnel syndrome was diagnosed and treated by surgery.

Ten months later the patient was admitted to our department because of persistence of his symptoms. Nerve conduction studies showed no response over the thenar muscle on median nerve stimulation at the wrist and elbow. Fibrillations and sparse motor unit potentials with increased duration and amplitude (up to 10 mV) were found in flexor digitorum superficialis muscle, in contrast to the normal Electromyography-findings of the right flexor carpi ulnaris. On median nerve stimulation at the elbow a low compound muscle action potential with an increased distal latency was recorded over flexor digitorum superficialis muscle in the right side (right: amplitude 1 mV, latency 9.0 ms, left: amplitude 7 mV, latency 3.2 ms). No sensory nerve action potential was recorded at the wrist on stimulation of the 1st, 2nd or 3^rd ^finger. Somatosensory evoked potentials of the left median and right radial and ulnar nerve were normal. No potential was recorded at Erb's point in the supraclavicular fossa, at the sixth and second cervical vertebra and the contralateral cortex on median nerve stimulation on the right.

**Figure 1:**
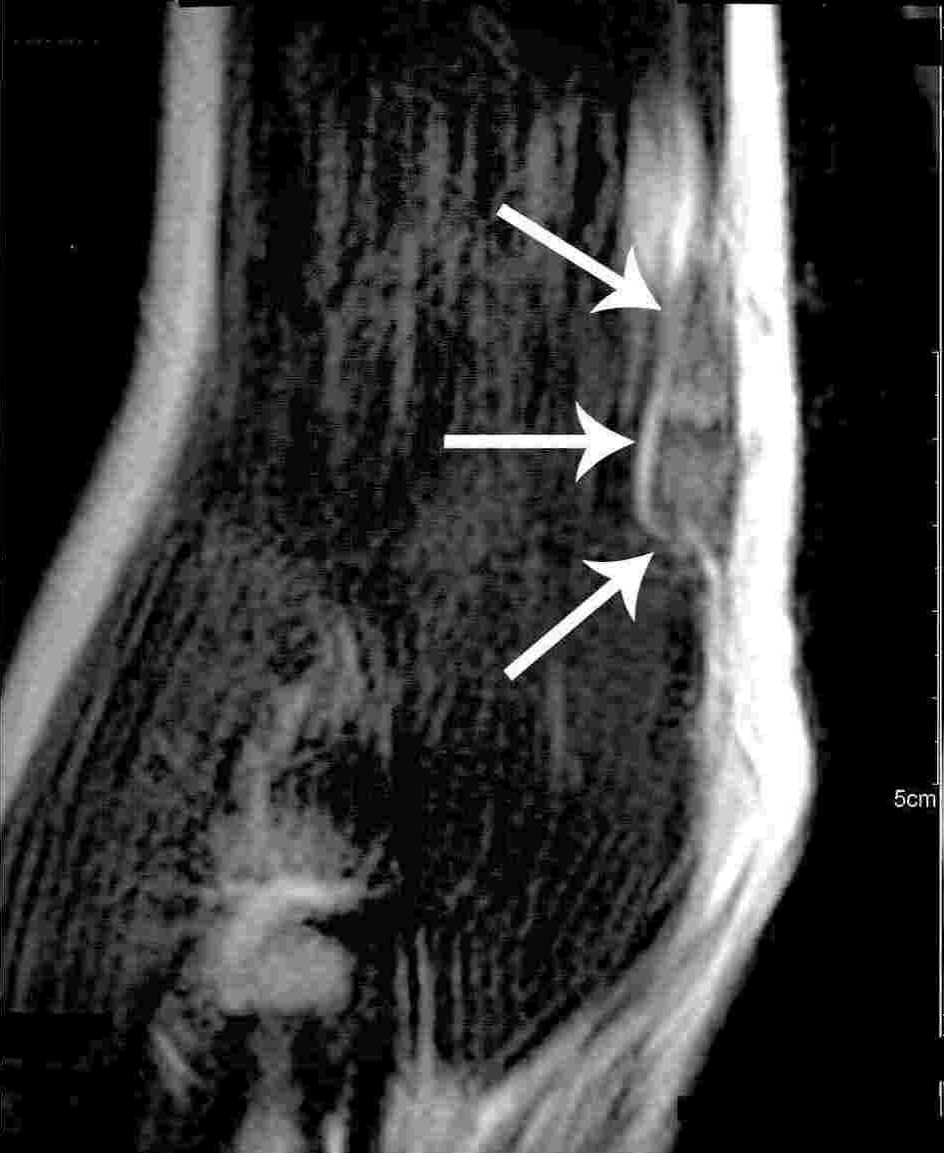
Coronal T2-weighted MRI reveals a slightly hyperintense fusiforme tumorous lesion of the median nerve approximately 5 cm above the right elbow (arrows).

**Figure 2:**
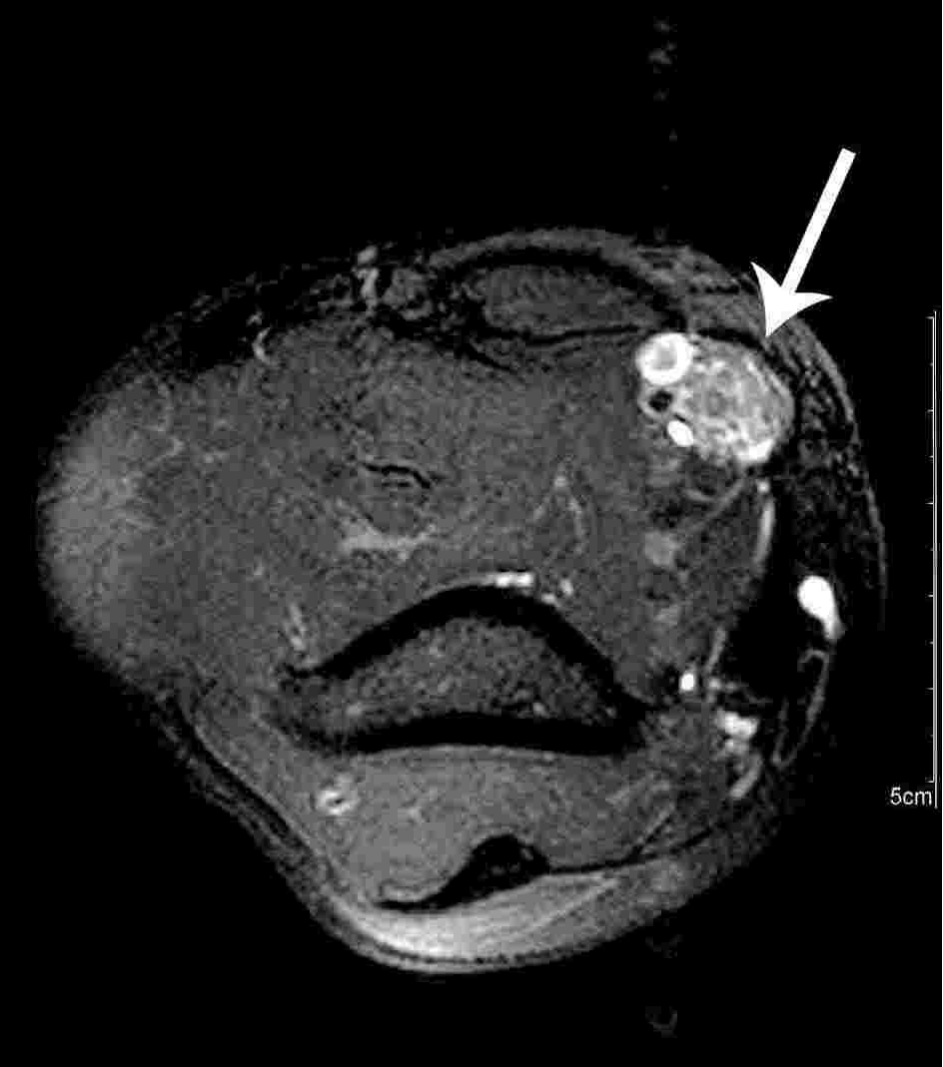
Axial fat-suppressed T2-weighted MRI shows a tumorous lesion of the median nerve with a fascicular pattern (arrow).

Proximal median nerve lesion was suggested. Palpation along the median nerve revealed a fusiform mass at the distal third of the right upper arm, which could be confirmed by MRI (see figure 1 and 2).

**Figure 3:**
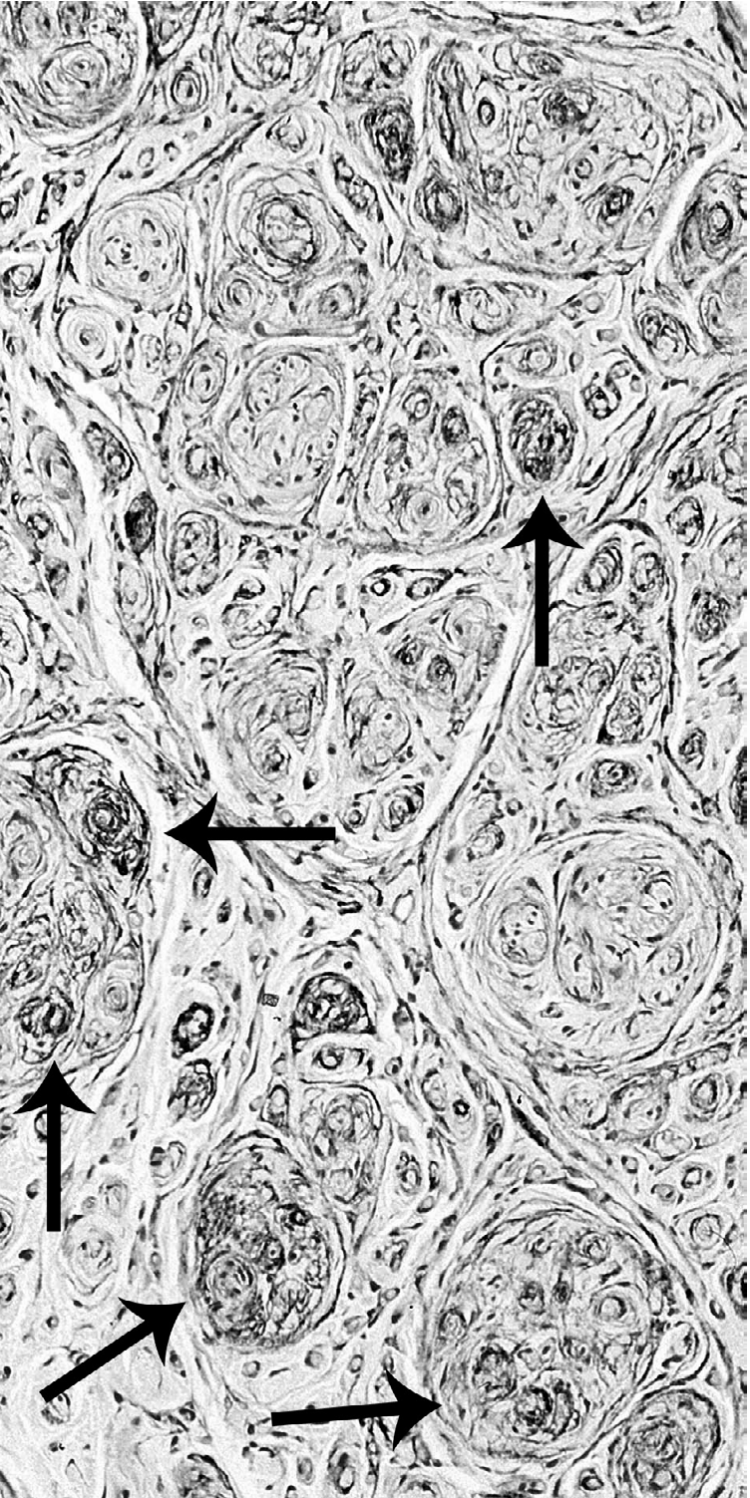
Multicompartment arrangement of concentric whirl-like formations showing strong immunoreactivity with the monoclonal antibody against the epithelial membrane antigen (arrows).

**Figure 4:**
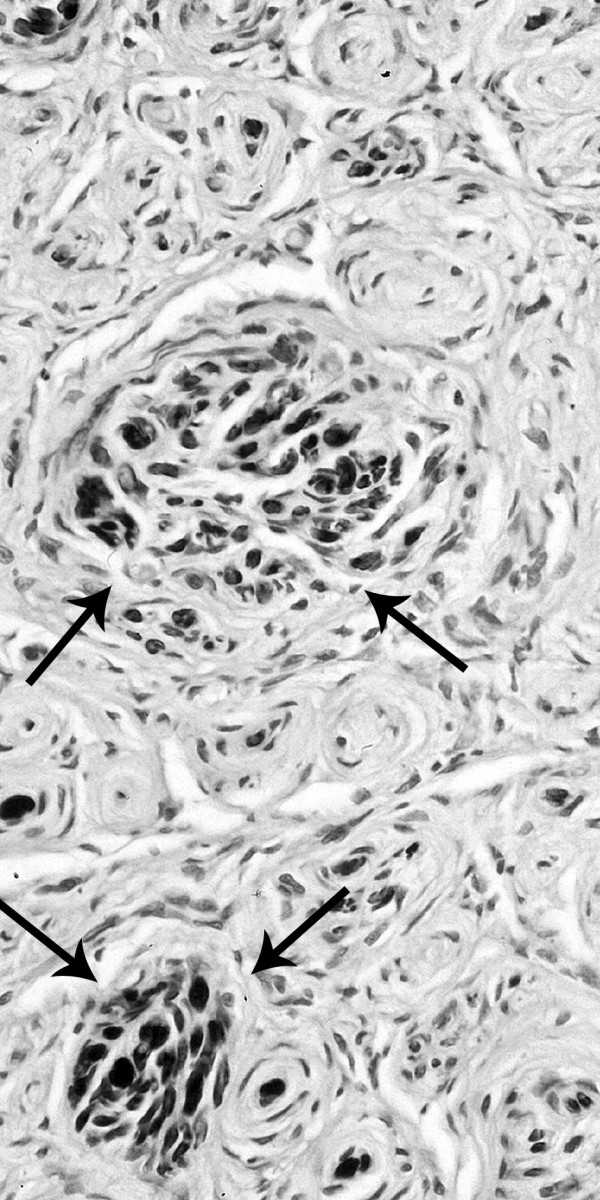
Immunohistochemistry using the antibody against protein S-100: The pseudo-onion bulb formations are S-100-negative. In addition, small groups of regenerating axonal sprouts (arrows) surrounded by a perineurial ensheathment are visible indicating some neuroma-like component.

Surgical resection of a 7 cm long segment of the median nerve with the tumorous lesion and replacement with a sural nerve graft was undertaken. Resection was necessary because of severe involvement of fascicles without any possibility to separate the tumour from the median nerve by micro-surgery. Histological investigation revealed marked peri- and endoneurial fibrosis, severe axonal loss as well as proliferation of concentric whorl-like formations resulting in multicompartment arrangement. These pseudo onion-bulb formations showed strong immunoreactivity for the epithelial membrane antigen and were predominantly negative for S-100 protein, suggesting a proliferation of perineurial cells rather than Schwann cells. In addition, small groups of regenerating axonal sprouts surrounded by perineurial ensheathment were visible, indicating some neuroma-like component (see figure 3 and 4).

The tumour was diagnosed as perineurioma and additional neuroma by histopathology. On clinical follow-up four years later a partial recovery of forearm muscle strength could be noted and the patient was free from pain.

## Conclusions

Carpal tunnel syndrome was falsely diagnosed in this case because of the increased distal latency in the median nerve, although during the first investigation an atrophy of the forearm muscle had already been noticed. Measurement of the median nerve latency to the atrophic flexor digitorum superficialis muscle would have disclosed the site of the lesion at that time.

Morphological and immunohistochemical studies classified the lesion as a mixed tumorous lesion with perineurioma and components of neuroma.

Neuromas are benign non-neoplastic lesions of the peripheral nerves which develop after disconnection of a nerve or a single fascicle. The history of severe pain with radiation to forearm and wrist immediately after procaine injection indicates that mechanical trauma by the needle and/or toxic effects of the local anesthetic may have caused nerve damage [14].

In perineuriomas, the underlying etiology still remains unclear. It has been recently suggested that perineuriomas are clonal neoplasms which may be associated with abnormalities on chromosome 22 [8]. In our case, however, hyperplastic reaction to the preceding nerve damage by trauma or toxic effect may have contributed to the pathogenesis of perineurioma and neuroma-like components, similar to mechanisms supposed to cause localized hypertrophic neuropathy [10,13]. Strong immunoreactivity for epithelial membrane antigen and virtually negative staining for S-100 unequivocally characterized the patient's tumor as a perineurioma and excluded localized hypertrophic neuropathy.

Perineurioma and localized hypertrophic neuropathy are characterized clinically by slowly progressive motor mononeuropathy without significant pain or numbness [2,10,15]. Neuromas, however, are painful. Pain and numbness in our patient were possibly caused by the neuroma-like component of the tumor.

## Authors' contributions

CS carried out the first neurological examination, study of literature and participated in writing and design of the manuscript. JEA made enquiries in Switzerland in order to get information about first injection and participated in writing of the manuscript. ENJ carried out the immunohistological investigations and prepared the figures 3 and 4. GS carried out the radiological investigations and prepared figures 1 and 2. LS performed the clinical follow-up, reviewed and corrected the manuscript. GA performed the nerve conduction studies and electromyography study and signed responsible for the description of this investigation.

## Competing interests

The author(s) declared that they have no competing interests.

## Pre-publication history

The pre-publication history for this paper can be accessed here:


